# Selective targeting of PPARγ by the natural product chelerythrine with a unique binding mode and improved antidiabetic potency

**DOI:** 10.1038/srep12222

**Published:** 2015-07-17

**Authors:** Weili Zheng, Lin Qiu, Rui Wang, Xuhui Feng, Yaping Han, Yanlin Zhu, Dezhou Chen, Yijie Liu, Lihua Jin, Yong Li

**Affiliations:** 1 State Key Laboratory of Cellular Stress Biology, Innovation Center for Cell Signaling Network, School of Life Scie-nces, Xiamen University, Fujian 361005, China

## Abstract

Type 2 diabetes mellitus (T2DM) is a pervasive metabolic syndrome that is characterized by insulin resistance, hyperglycemia and dyslipidemia. As full agonists of PPARγ, thiazolidinedione (TZD) drugs elicit antidiabetic effects by targeting PPARγ but is accompanied by weight gain, fluid retention and cardiovascular risk associated with their transcriptional agonism potency. We here identify a natural product chelerythrine as a unique selective PPAR modulator (SPPARM) with a potent PPARγ binding activity but much less classical receptor transcriptional agonism. Structural analysis reveals that chelerythrine exhibits unique binding in parallel with H3 of PPARγ. Unlike TZDs, chelerythrine destabilizes helix 12, especially residue tyrosine 473, resulting in a loose configuration of AF-2 and a selective cofactor profile distinct from TZDs, leading to a differential target gene profile in adipogenesis in *db/db* diabetic mice. Moreover, chelerythrine improved insulin sensitivity by more potently blocking the phosphorylation of PPARγ by CDK5 compared to TZDs. These data fundamentally elucidate the mechanism by which chelerythrine retains the benefits of improving insulin sensitivity while reducing the adverse effects of TZDs, suggesting that the natural product chelerythrine is a very promising pharmacological agent by selectively targeting PPARγ for further development in the clinical treatment of insulin resistance.

Nuclear receptors (NRs) are crucial transcriptional factors controlling gene expression that function as regulative proteins that bind to specific sequences of the corresponding response elements in the vicinity of their target genes^1^,^2^,^3^. The binding of various ligands to the ligand-binding domain of NRs within flexible pockets reflects a common structural property that represents an ideal drug target in conjunction with the pleiotropic roles of NRs in differentiation, inflammation and metabolic homeostasis control^4^,^5^.

Peroxisome proliferator-activated receptor gamma (PPARγ, NR1C3), a member of the NR family, is highly expressed in adipose tissues and plays an indispensable role in adipocyte differentiation^6^. Paradoxically, the most notable pharmacological role of PPARγ ligands is their ability to improve insulin sensitivity, hyperglycemia and dyslipidemia in type 2 diabetes mellitus^7^,^8^. In fact, some dietary controls of type 2 diabetes have been associated with PPARγ modulations^9^,^10^. As to pharmacological intervention, TZDs play indispensable roles as PPARγ full agonists in treating T2DM syndrome and are described as insulin sensitizers^11^. TZDs form strong hydrogen bonds with tyrosine 473 in helix 12 of PPARγ, which stabilizes AF2 and also directly correlates with full agonism^12^,^13^. However, TZDs treatment results in adverse effects of classical PPARγ agonists, including obesity and weight gain, which are pervasive among diabetes patients, as well as fluid retention and cardiovascular risk^14^. As a result, attention has been focused on another type of PPARγ ligands, selective PPAR modulators (SPPARMs)^15^,^16^, which, unlike TZDs, do not exhibit full agonism. Furthermore, SPPARMs, such as MRL-24 and SR1664^17^,^18^, are also effective in insulin sensitization. In addition, selective modulators of PPARγ have a more dynamic effect on the conformation of AF-2 than TZD full agonists^19^, this conformational change also results in differential cofactors profiling^20^. Conclusively, transactivity is not the direct mechanism underlying the PPARγ antidiabetic potency. Moreover, TZDs and SPPARMs such as MRL-24, SR1664 and UHC1 all inhibit the CDK5-mediated phosphorylation of PPARγ at serine 273^21^,^22^. Consequently, discovering PPARγ selective modulators with partial agonism that exert diabetic effects similar to rosiglitazone while avoiding its side effects is significant.

*Chelidonium majus* (greater celandine) has been used in medical therapy as an alkaloid-containing plant since ancient times and exhibits antiviral, antitumour, antibacterial, antifungal and anti-inflammatory effects^23^,^24^. Chelerythrine is a major representative of quaternary benzophenanthridine alkaloids (QBAs) in *C. majus* and known to be a PKC inhibitor^25^. In this study, we present comprehensive structural and functional evidence for identification chelerythrine as a selective PPARγ modulator that potently inhibited CDK5-mediated phosphorylation of PPARγ. In biochemical assays, chelerythrine directly and potently binds to the PPARγ LBD. In addition, we demonstrated that the unique binding of chelerythrine relative to TZDs resulted in a differential cofactor profile and partial agonism. Structurally, there are also conformational changes after chelerythrine binding to PPARγ relative to rosiglitazone, especially with respect to helix 3, helix 7 and helix 11, which indirectly contributes to the dynamics of AF-2. Moreover, we investigated the antidiabetic effects of chelerythrine in high-fat diet *db/db* and KKAy diabetic mice, which demonstrated that chelerythrine improved metabolic parameters and insulin sensitivity without weight gain. The gene profiling in adipose tissue in our study further confirmed that chelerythrine did not enhance adipogenesis like rosiglitazone did. In conclusion, chelerythrine exhibits greater potency in regulating glucose homeostasis through inhibiting CDK5-mediated PPARγ phosphorylation than do TZDs and may represent a novel pharmacological agent in treating metabolic disease associated with T2DM.

## Results

### Identification of chelerythrine as a novel PPARγ selective modulator with high binding potency but weak classical agonism

**Figure 1:**
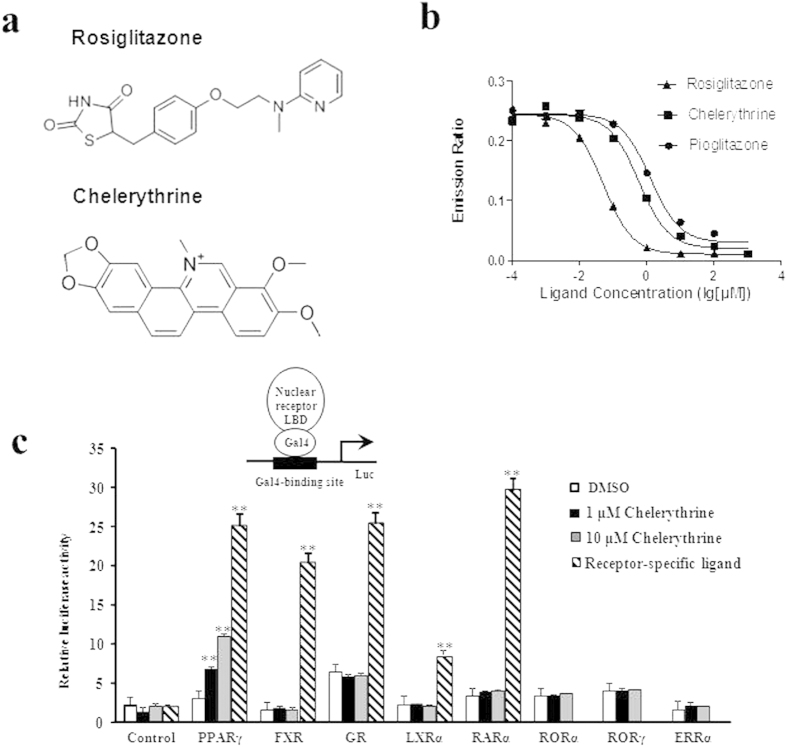
Identification of chelerythrine as a PPARγ selective modulator. (**a**) Chemical structures of chelerythrine and rosiglitazone. (**b**) The binding affinity of chelerythrine to PPARγ in the Lantha Screen assay. Triangles, rosiglitazone; squares, chelerythrine; dots, pioglitazone. (**c**) Receptor-specific transactivation by chelerythrine. 293T cells were cotransfected with the pG5Luc reporter together with the plasmids encoding various nuclear receptor LBDs fused with the Gal4 DNA-binding domain. After transfection, cells were treated with DMSO (white bars), 1 μmol/l (black bars) and 10 μmol/l (gray bars) chelerythrine or ligands specific for each receptor(stripped bars): PPARγ, 1 μmol/l rosiglitazone; FXR, 0.1 μmol/l GW4064; GR, 0.1 μmol/l dexamethasone; LXRα, 1 μmol/l T0901317; RARα, 1 μmol/l all-trans retinoid acid. Error bars represent s.e.m.; *p < 0.05, **p < 0.01, compared with vehicle.

Considering the relationship between PPARγ and insulin resistance, we performed a high-throughput AlphaScreen™ assay, which determines the efficacy of small molecules in influencing binding affinity of PPARγ with coactivator peptides^26^. Results from the AlphaScreen™ revealed that a type of QBA-chelerythrine weakly stimulated the binding affinity of PPARγ LBD and its coactivators. Notably, the chemical structure of chelerythrine exhibits a distinct scaffold from that of rosiglitazone (Fig. 1a), which indicates that it may exhibit different activities. To confirm the binding potency of chelerythrine to PPARγ, we used a Lantha TR-FRET competitive binding assay to compare the half-maximum inhibitory concentration (IC50) of chelerythrine and rosiglitazone. The IC50 of chelerythrine is 566 nM (Fig. 1b), which is ten-fold greater than that of rosiglitazone but less than that of another TZD, pioglitazone. Furthermore, in the transactivity assay, we used a GAL-4 driven reporter to determine the specificity and transactivity of chelerythrine. In various highly conserved NR LBDs, chelerythrine specifically activated PPARγ with a low transactivity distinct from rosiglitazone (Fig. 1c and Supplementary Figure 1). Interestingly, as Supplementary Figure 2 indicated, the 10 μM of chelerythrine did not induce any adipogenesis, which was similar with the vehicle control, while 10 μM of rosiglitazone induced obvious differentiated adipocytes, demonstrating that the adipogenic activity of chelerythrine is much weaker than rosiglitazone (Supplementary Figure 2). Collectively, these data indicate that chelerythrine is a bona fide PPARγ selective modulator with high binding potency but weak classical agonism.

### Chelerythrine modulates PPARγ cofactor profiling

One characteristic of PPARγ ligands is that they regulate different target gene groups by regulating the recruitment and release of different cofactors^27^. When binding to PPARγ LBD, agonists induce coactivator recruitment and corepressor dissociation, whereas antagonists behave in the opposite way. These cofactors bind to the nuclear receptor complex, modify the basic chromatin structure and sequentially modulate transcriptional machinery to regulate the target genes.

**Figure 2:**
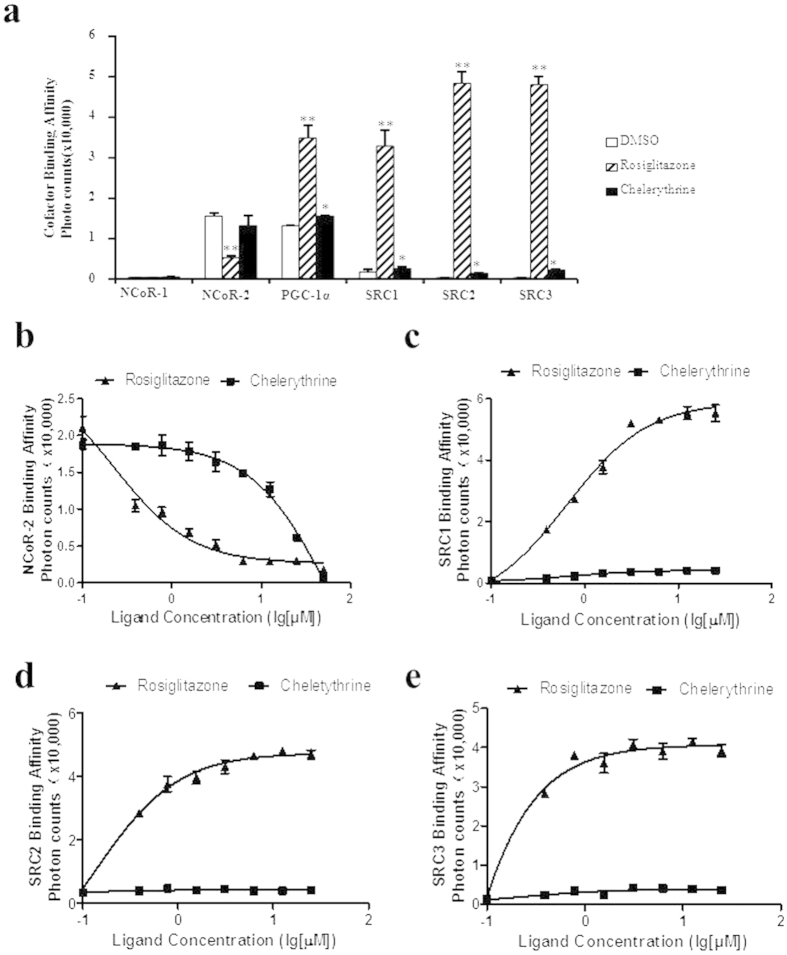
Differential cofactor binding interactions of chelerythrine to PPARγ LBD by AlphaScreen assay. (**a**) Binding of various cofactors interaction motifs to the PPARγ LBD in response to 1 μmol/l rosiglitazone and 5 μmol/l chelerythrine. The background reading with the PPARγ LBD is less than 300 photon counts. (**b–e**) Dose curve of rosiglitazone and chelerythrine in recruiting the coactivators SRC1, SRC2 or SRC3 peptides and release of corepressor NCoR-2 peptide. The peptide sequences are listed in experimental procedures. Error bars represent s.e.m.; *p < 0.05, **p < 0.01, compared with vehicle.

We then used the AlphaScreen™ assay to determine the ability of chelerythrine to modify the cofactors profiling of PPARγ. As Fig. 2a demonstrates, chelerythrine recruited the coactivator peptides from SRC1, SRC2, SRC3 and PGC-1α to PPARγ more weakly than rosiglitazone. Interestingly, with respect to its corepressor activity, chelerythrine slowed the dissociation of NCoR-2, a corepressor peptide from NCoR, compared to rosiglitazone treatment (Fig. 2a). Furthermore, we used a gradient concentration of ligands to demonstrate that chelerythrine is substantially less effective in recruiting SRC family coactivators and dissociating the NCoR-2 corepressor (Fig. 2b–e). Our study indicates that chelerythrine is a PPARγ selective modulator with a cofactor profile that differs from rosiglitazone, which leads to its low transcriptional activity and partial agonism.

### Chelerythrine recognizes PPARγ in a unique binding mode

**Figure 3:**
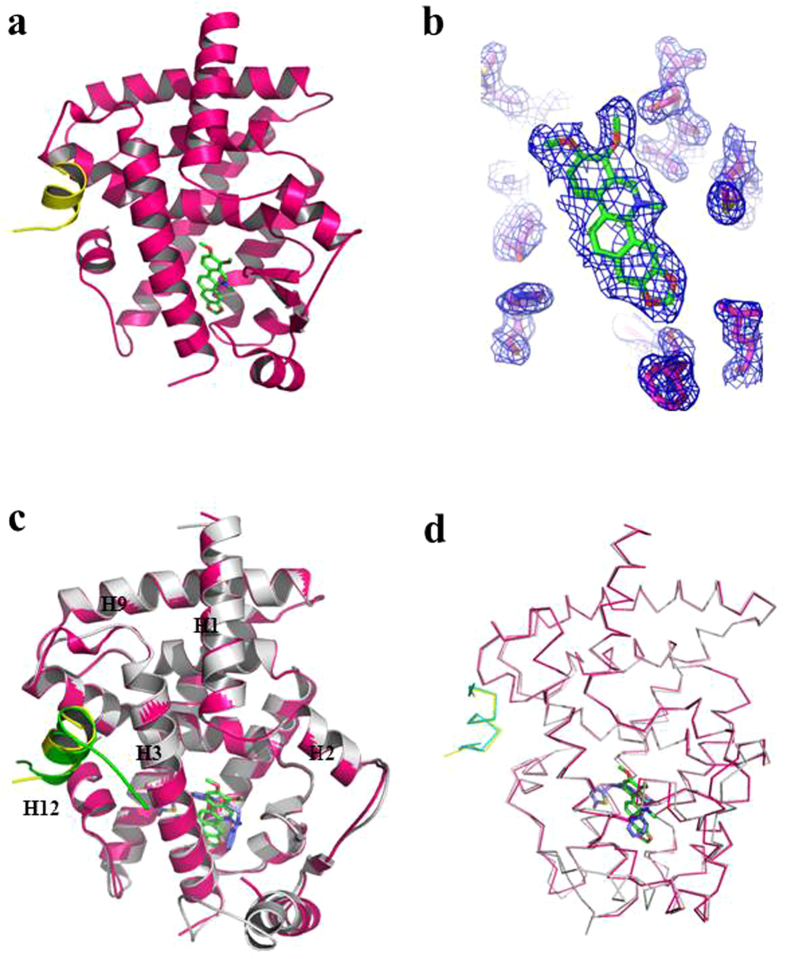
The structure of the PPARγ LBD complexed with chelerythrine. (**a**) The structure of the PPARγ LBD bound with chelerythrine in a ribbon representation. PPARγ LBD is colored in hot pink, and the SRC1 motif is in yellow. Chelerythrine is shown as a stick representation with carbon and oxygen atoms depicted in green and red, respectively. (**b**) 2Fo-Fc electron density map (1.0 σ) demonstrated chelerythrine bound to the PPARγ LBD. (**c,d**) Alignment of PPARγ/chelerythrine (pink) with PPARγ/rosiglitazone (gray) in ribbon and stick representation separately. Chelerythrine is shown as green structure and rosiglitazone is marine.

**Figure 4:**
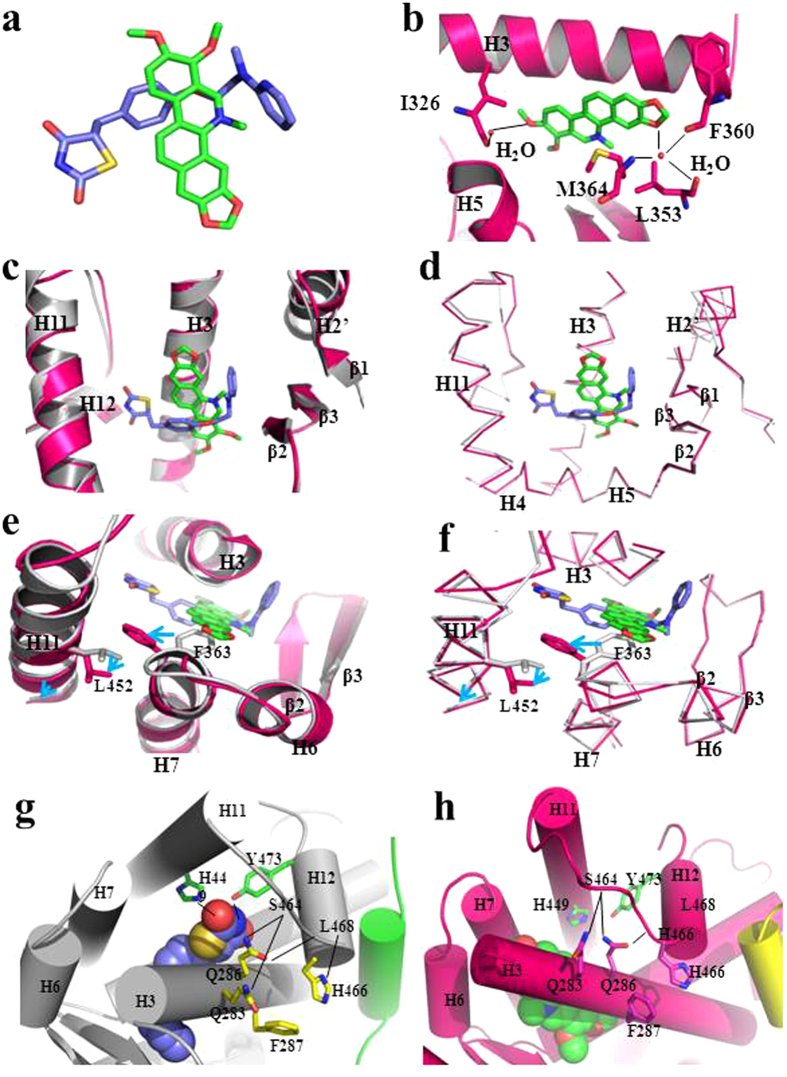
Chelerythrine complexed with PPARγ in a unique binding interaction as a selective modulator. (**a**) Superimposition of chelerythrine structure (green) with the rosiglitazone structure (marine). (**b**) The indirect hydrogen bond between chelerythrine and PPARγ is shown as black stick. Chelerythrine is shown as green structure;PPARγ is shown as hot pink ribbon representaion; water is shown as pink dots. (**c,d**) Conformational changes of PPARγ induced by chelerythrine relative to rosiglitazone. PPARγ/chelerythrine (pink) with PPARγ/rosiglitazone (gray) in ribbon and stick representation separately. (**e,f**) Conformational changes (indicated by arrows) of the residues side chains in the PPARγ pocket involved in chelerythrine binding. (**g,h**) Analysis of chelerythrine interaction with PPARγ H12 distinct from rosiglitazone.

To further understand the molecular mechanism underlying chelerythrine interaction with PPARγ, we solved the crystal structure of PPARγ complexed with chelerythrine at a resolution of 1.98 Å (Fig. 3a and Supplementary Table 1)^28^. The structure reveals that chelerythrine bound PPARγ LBD adopts a classical three-layer helical sandwich structure^29^, which is globally the same as almost all NR LBDs. In the electron-density map presented in Fig. 3b, chelerythrine clearly binds in the PPARγ LBD. Although structural alignment of PPARγ/chelerythrine and PPARγ/rosiglitazone reveals the whole structure of chelerythrine-bound PPARγ is conserved relative to that of rosiglitazone^30^ (Fig. 3c,d), the binding of chelerythrine gives rise to several changes in the PPARγ ligand-binding pocket (LBP). As a selective modulator with weaker transactivity, chelerythrine exhibits a nearly flat scaffold that is structurally distinct from rosiglitazone (Fig. 4a). Both ligands docking in a similar binding site in the PPARγ LBP, whereas the two small molecules orient in a perpendicular manner (Fig. 4c,d).

The interactions that ligands anchor in the LBP vary between PPARγ/chelerythrine and PPARγ/rosiglitazone. Chelerythrine is docked in the LBP mainly through reversible hydrophobic interactions and water-mediated contacts. There are two indirect hydrogen bond interactions through H_2_O molecules (Fig. 4b). First, the carbonyl oxygen of the amide from Ile326 makes a hydrogen bond contact with methoxy in chelerythrine through H_2_O molecule. On the other side, the nitrogen or carbonyl oxygen of an amide from Leu353, Phe360 and Met364 make additional hydrogen bond contacting to the oxygen of heterocyclic chelerythrine through H_2_O molecule.

### Structural insights into the partial agonism of chelerythrine

The cofactor profiling assay in our study indicated that chelerythrine induces conformation changes in the PPARγ AF-2 surface to create a new cofactor binding pattern. Notably, AF-2 also correlates with the degree of ligand transactivation. Therefore, we further dissect the conformation changes induced by chelerythrine in the AF-2 surface to explain high-affinity PPARγ ligands with differential agonism. As shown in Fig. 4g, rosiglitazone forms a hydrogen bond with Tyr473 and His449 through its nitrogen and oxygen atom in TZD group, which further allows helix 12 to dock against helix 3 and helix 11. However, this strong correlation is absent in the PPARγ/chelerythrine complex (Fig. 4h), which is the direct evidence of the partial agonism of chelerythrine. Within the PPARγ LBP, chelerythrine lies parallel to helix 3 (Fig. 4c,d), which is in a perpendicular orientation from rosiglitazone. Together, helix 7 and helix 11 also shift outwardly in response to the perpendicular orientation of chelerythrine relative to rosiglitazone. As a result, the hydrophobic benzene ring side chain of Phe363 shifts externally from its rosiglitazone-bound conformation, driving the side chain of Leu452 outward even as the major conformation of helix 11 is conserved as in PPARγ/rosiglitazone (Fig. 4e,f). Collectively, these results suggest that the selective modulator chelerythrine binds PPARγ to induce dynamic changes in the AF-2 surface, leading to differential cofactor profiling and partial agonism.

### Chelerythrine improves insulin resistance without weight gain in *db/db* mice

**Figure 5:**
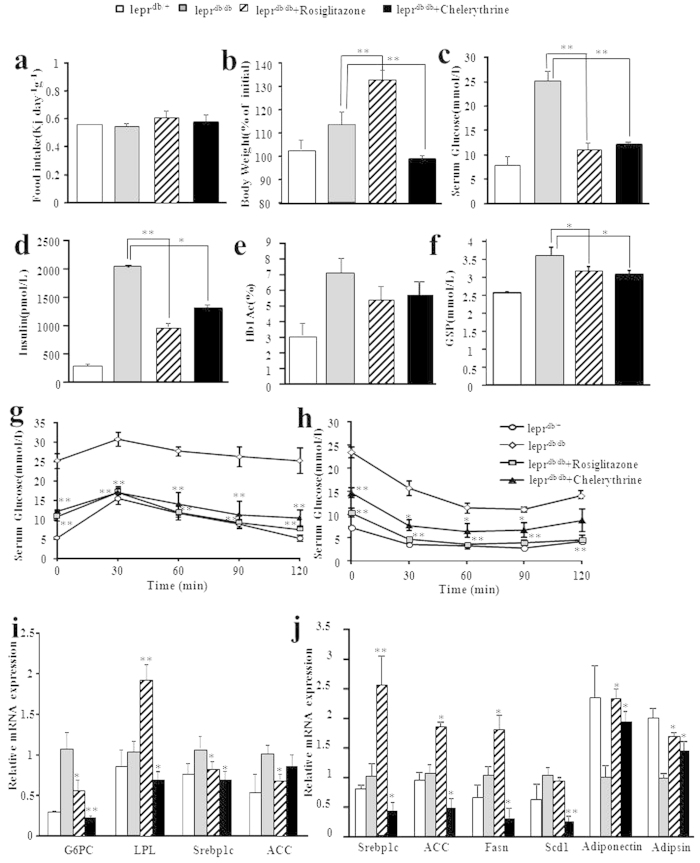
Chelerythrine improved glucose tolerance and insulin sensitivity with reduced adipogenesis activity in *db/db* mice. The food intake (**a**) body weight (**b**) serum glucose levels (**c**) serum insulin (**d**) Hb1Ac (**e**) and GSP (**f**) were measured in *db/db* mice and their littermate controls (white bars or round symbols). *db/db* mice were i.p. injected with vehicle (HBC, grey bars or diamond symbols), 3 mg/kg rosiglitazone (Rosi, stripped bars or square symbols) or 3 mg/kg chelerythrine (CHE, black bars or triangle symbols) for 14 days. Insulin (1 unit/kg) and glucose (1 g/kg), respectively, were administered by i.p. injected in 6-h-fasted *db/db* mice or their littermate controls for the GTT (**g**) and ITT (**h**). (**i,j**) The mRNA levels of genes involved in adipogenesis and glucose metabolsim in liver (**i**) and epididymal fat pads (**j**) were measured. Error bars represent s.e.m.; *p < 0.05, **p < 0.01, compared with vehicle treated *db/db* mice.

As PPARγ is a significant drug target for metabolic syndrome associated with obesity-induced insulin resistance and hyperglycemia^31^,^32^, we used the *db/db* diabetic mouse model and their littermates to determine whether chelerythrine has the ability to improve glucose homeostasis in these mice. Treating *db/db* mice for 14 days with 3 mg/kg rosiglitazone or 3 mg/kg chelerythrine, we determined that rosiglitazone increased body weight significantly by approximatedly 30% compared to vehicle control with the equal food intake (Fig. 5a), whereas the body weight of mice treated with chelerythrine kept stable (Fig. 5b), demonstrating that as a selective modulator, chelerythrine did not cause the weight gain side effects that rosiglitazone did. In consist with this weight control advantages, the liver and epididymal fat pads weight of chelerythrine treated *db/db* mice were both obviously lower than that of vehicle and rosiglitazone treated mice (Supplementary Figure 3a & 3b), which is also supported by that the expression of lipogenesis related genes Srebp-1c, Fasn, Scd1, etc. in liver and epi-WAT are significantly downregulated after chelerythrine treatment compared to rosiglitazone (Fig. 5i,j). Of note, we have measured the activity of the serum alanine aminotransferase (ALT) of chelerythrine-treated mice, and chelerythrine did not cause liver toxicity in diabetic mice (Supplementary Figure 3d). Moreover, the genes expression in mice liver, such as the downregulation of the gluconeogenesis enzyme G6Pase, elucidated the molecular mechanism of the glucose metabolism regulated by chelerythrine (Fig. 5i). Meanwhile, fasted metabolic parameters such as serum glucose, insulin and cholesterol were all downregulated by both chelerythrine and rosiglitazone treatment (Fig. 5c,d and Supplementary Figure 3c). As gold markers in clinical diagnostic, hemoglobin A1c (Hb1Ac) and glycosylated serum protein (GSP) were both downregulated after chelerythrine and rosiglitazone treatment (Fig. 5e,f). Our study also indicated that the treatment of chelerythrine downregulated CD36 and IL-1β as well as inflammation related genes like IFNγ and TNFα, which are of great benefits for the treatment of diabetes (Supplementary Figure 4).

We further performed a glucose tolerance test (GTT) and insulin tolerance test (ITT) to determine the insulin sensitivity. As shown in Fig. 5g,h, chelerythrine and rosiglitazone both improved the glucose tolerance and insulin sensitivity efficaciously in diabetic mice, which is even as senstive as their littermate controls. Furthermore, the effect of chelerythrine in improving insulin sensitivity were consistent in another diabetic mouse model, KKAy, which indicated the same potency of chelerythrine in mediating glucose homeostasis (Supplementary Figure 5). Collectively, these data indicate that chelerythrine is as effective as rosiglitazone in improving insulin sensitivity and homeostasis of glucose but without any side effects of weight gain.

### Chelerythrine blocks CDK5-mediated phosphorylation of PPARγ and inhibits adipogenesis

**Figure 6:**
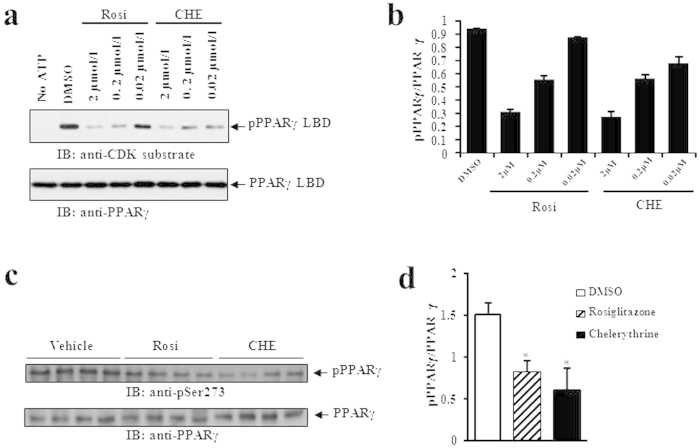
PPARγ modulator chelerythrine is an insulin sensitizer that blocks the CDK5-mediated phosphorylation of PPARγ at Ser273 *in vitro* and *in vivo*. (**a**) *In vitro* CDK5 assay on PPARγ LBD incubated with gradient concentration of rosiglitazone or chelerythrine. (**b**) Quantification of PPARγ phosphorylation relative to total PPARγ *in vitro*. (**c**)* In vivo* phosphorylation of PPARγ was analyzed in epididymal fat pads of mice with chelerythrine treatment as shown in Fig. 5. (**d**) Quantification of PPARγ phosphorylation relative to total PPARγ *in vivo*. IB, immunoblot; pPPARγ, phosphorylated PPARγ; pSer273, phosphorylated PPARγ at Ser273; Rosi, rosiglitazone; CHE, chelerythrine.

The selective PPARγ modulator chelerythrine exhibited effects in maintaining glucose metabolism, although its transcriptional activity is considerably low. It was reported that inhibition of CDK5-mediated phosphorylation of PPARγ is related to insulin sensitivity^17^. We used the *in vitro* and *in vivo* CDK5 kinase assay to determine whether the insulin sensitizer chelerythrine retains the ability to inhibit CDK5-mediated phosphorylation of PPARγ S273. As shown in Fig. 6a,b, chelerythrine blocked the phosphorylation of PPARγ serine 273 by CDK5 in a concentration-dependent manner. More importantly, chelerythrine is more effective in the inhibition of PPARγ phosphorylation than rosiglitazone despite its weak transcriptional activity *in vitro*. Consistent with the data *in vitro*, both rosiglitazone and chelerythrine caused a similar reduction in PPARγ phosphorylation at Ser273 in mice white adipose tissues (Fig. 6c,d).

CDK5-mediated phosphorylation of PPARγ is associated with obesity, and dysregulated the expression of a subset of PPARγ-regulated genes, including the adiponectin(*Adipoq*) and adipsin, which were significant in PPARγ-mediated insulin sensitivity^33^,^34^. As the data shown in the analyses of the gene expression of adipose tissue in obese mice (Fig. 5j), adiponectin and adipsin were significantly upregulated by chelerythrine treatment, which was helpful in improvement of fat oxidation and insulin resistance.

## Discussion

Chelerythrine is a major representative of quaternary benzophenanthridine alkaloids (QBAs) in *C. majus*, and the effects of chelerythrine in medical therapy contribute to antiviral, antitumour, antimicrobial and anti-inflammatory activities^23^,^24^. In this study, we found that chelerythrine is a modulating ligand for PPARγ by a high-throughput screen, thereby uncovering a novel signaling route for this QBA molecule. The results from both the biochemical AlphaScreen™ assay and cell-based reporter assay revealed that chelerythrine was a partial agonist for PPARγ due to its weak ability to recruit coactivators and activate the transcriptional activity of PPARγ compared to the typical full agonist rosiglitazone.

Recent studies highlight that the binding of ligands to PPARγ can block the CDK5-mediated phosphorylation of PPARγ at Ser273 by the Spiegelman group, which is also tightly associated with the anti-diabetic effects of these ligands^17^. Accordingly, ligands with high inhibitory effects on PPARγ phosphorylation by CDK5 that lack classical agonism are optimal drug candidates for diabetes. Since a significant consequence of the transcriptional activation of PPARγ is induction of adipocyte differentiation, a major factor leading to the adverse effects of PPARγ ligands. In our study, chelerythrine exhibited powerful anti-diabetic effects similar to TZDs, whose potency correlates very well with its ability to block CDK5-mediated phosphorylation of PPARγ, supporting the critical roles of PPARγ phosphorylation by CDK5 in improving insulin resistance by its ligands. Strikingly, the same research team recently found an alternative way of phosphorylating PPARγ through the extracellular signal-regulated (ERK) kinases, which is also correlated with insulin resistance, thereby providing another evidence for the significance in modulating the phosphorylation of PPARγ and also another promising drug target in treatment of T2DM^35^.

Our results demonstrate that chelerythrine exhibits several key features distinct from the full agonist rosiglitazone. First, the scaffold of chelerythrine is distinct from TZDs and is nearly flat, thus serving as an alternative template for designing drugs targeting PPARγ. Second, the specific interactions between the critical LBD residues of PPARγ and chelerythrine provide critical perspectives and novel pharmacological binding mode in designing selective PPARγ modulators. Our structural observations found that chelerythrine interacts with helix 3 in a parallel manner like but not the same with other partial agonists, whereas the interaction is perpendicular to that of rosiglitazone. This significant discrepancy causes serial conformational changes in the PPARγ LBD, including in helix 3, helix 7 and helix 11. These subtle shifts significantly influence helix 12, as the differential orientation of chelerythrine and its global chemical structure make it impossible to form the hydrogen bond with Tyr473 in helix 12 to further stabilize AF-2. Thus, chelerythrine lacks the most significant feature of full PPARγ agonists. Taken together, the unique characteristics of chelerythrine may represent new pharmacophores that can be optimised for selectively targeting PPARγ.

Among PPARγ selective modulators, chelerythrine also exhibits a distinct binding interaction with PPARγ. Many other partial agonists, such as MRL-24 and SR1664, make contact with helix 3 and β sheets instead of stabilizing helix 12. Lacking those contacts, most of the chelerythrine interactions with PPARγ are mediated through hydrophobic contacts and a few weak hydrogen bonds, which may underlie the unique weak agonism of chelerythrine. Furthermore, distinct AF-2 conformational dynamics induces by rosiglitazone and chelerythrine influence cofactor profiling. For example, SRC family peptides bound more potently to PPARγ induced by rosiglitazone than chelerythrine. In the contrary, rosiglitazone released NCoR-2 peptide more readily than chelerythrine. Thus, these unique structural binding interactions and selective cofactor profiles suggest that chelerythrine could be a lead compound in designing pharmacophores with weak agonism targeting PPARγ.

As a partial agonist, chelerythrine retains the beneficial effects of rosiglitazone but lacks the side effect of weight gain, which is largely related to PPARγ adipogenic activity through transactivity. In the chelerythrine-treated mice, the expression of adipogenic genes was downregulated relative to rosiglitazone-treated mice. Furthermore, some dysregulated genes associated with obesity were correctly regulated by chelerythrine, including adiponectin and adipsin, which are dysregulated downstream of CDK5-mediated PPARγ phosphorylation. Moreover, chelerythrine can potently block the CDK5-mediated phosphorylation of PPARγ to a greater degree than rosiglitazone. Taken together, as a PPARγ modulator, chelerythrine improved not only the homeostasis of glucose metabolism, but also that of the lipid metabolism, suggesting the natural product chelerythrine is a very promising pharmacological agent by selectively targeting PPARγ for further development in the clinical treatment of insulin resistance.

## Methods

### Protein preparation

Human PPARγ LBD (residues 206–477) was expressed as an N-terminal 6xHis fusion protein from the expression vector pET24a (Novagen, Germany). BL21(DE3) cells transformed with the expression plasmid were grown in LB broth at 25 °C to an OD600 of approximately 1.0 and induced with 0.1 mmol/l isopropyl 1-thio-β-D-galactopyranoside (IPTG) at 16 °C. Cells were harvested and sonicated in 200 ml of extract buffer (20 mmol/l Tris pH 8.0, 150 mmol/l NaCl, 10% glycerol, and 25 mmol/l imidazole) per 6 liters of cells. The lysate was centrifuged at 20,000 rpm for 30 min, and the supernatant was loaded on a 5-ml NiSO_4_-loaded HiTrap HP column (GE Healthcare, PA, USA). The column was washed with extract buffer, and the protein was eluted with a gradient of 25–500 mmol/l imidazole. The PPARγ LBD was further purified with a SP-Sepharose column (GE Healthcare, PA, USA). To prepare the protein-ligand complex, 5-fold excess of the chelerythrine (Sigma, USA) and 2-fold excess of steroid receptor coactivator 1 (SRC1) peptide (AQQKSLLQQLLTE) were added to the purified protein, followed by filter concentration to 10 mg/ml.

### Crystallization, data collection and structure determination

The crystals of the PPARγ/chelerythrine complex were grown at room temperature in hanging drops containing 1.0 μl of the above protein-ligand solutions and 1.0 μl of well buffer containing 0.2 mol/l sodium thiocyanate and 20% w/v polyethylene glycol 3350. The crystals were directly flash frozen in liquid nitrogen for data collection. The observed reflections were reduced, merged and scaled with DENZO and SCALEPACK in the HKL2000 package. The structures were determined by molecular replacement in the CCP4 suite (http://www.ccp4.ac.uk)^36^. Manual model building was carried out with Coot^37^, followed by REFMAC refinement in the CCP4 suite.

### Cofactor binding assays

The binding of various coregulator peptide motifs to PPARγ LBD in response to ligands were determined by AlphaScreen™ assays using a hexahistidine detection kit from Perkin-Elmer as described^26^. The experiments were conducted with approximately 20–40 nmol/l receptor LBD and 20 nmol/l biotinylated coregulator peptides in the presence of 5 μg/ml donor and acceptor beads in a buffer containing 50 mmol/l MOPS, 50 mmol/l NaF, 0.05 mmol/l CHAPS, and 0.1 mg/ml bovine serum albumin, all adjusted to a pH of 7.4.

The peptides with an N-terminal biotinylation are listed below.

SRC1, SPSSHSSLTERHKILHRLLQEGSP;

SRC2, QEPVSPKKKENALLRYLLDKDDTKD;

SRC3, PDAASKHKQLSELLRGGSG;

PGC-1α, AEEPSLLKKLLLAPA;

NCoR-1, QVPRTHRLITLADHICQIITQDFAR;

NCoR-2, GHSFADPASNLGLEDIIRKALMGSF.

### Transient transfection assay

293T cells(ATCC, USA) were maintained in DMEM containing 10% fetal bovine serum (FBS) and were transiently transfected using Lipofectamine 2000 reagent (Invitrogen, USA). All mutant PPARγ plasmids were created using the Quick-Change site-directed mutagenesis kit (Stratagene, USA). Twenty-four-well plates were plated 24 hours prior to transfection (5 × 10^4^ cells per well). For the Gal4-driven reporter assays, the cells were transfected with 200 ng of Gal4-LBDs of various nuclear receptors and 200 ng of pG5Luc reporter (Promega, USA). Ligands were added five hours after transfection. Cells were harvested 24 h later for the luciferase assays. Luciferase activities were analyzed as the the instruction of CheckMate™ Mammalian Two-Hybrid System (Promega, USA).

### *In vitro* kinase assay

The *in vitro* CDK kinase assay was performed as previously described^18^. Briefly, 1μg purified His-tagged PPARγ LBD (residues 206–477) was incubated with 50 ng active CDK5/p25 (Invitrogen, USA) in assay buffer (25 mmol/l Tris pH7.5, 10 mmol/l MgCl_2_, 5 mmol/l β-glycerophosphate, 0.1 mmol/l Na_3_VO_4_, 2 mmol/l DTT) (Cell Signaling Technology, USA) containing 100 μmol/l ATP in a 50 μl reaction for 30 min at room temperature. PPARγ ligands were preincubated with PPARγ LBD protein for 30 min before the assay was performed. Phosphorylation of the PPARγ LBD was analyzed by western blotting with an anti-CDK substrate antibody (Cell Signaling Technology, USA).

### Preparation of white adipose tissue lysates and immunoblotting

White adipose tissues from mice treated with compounds were homogenized in RIPA buffer (50 mmol/l Tris pH7.5, 150 mmol/l NaCl, 1% NP-40, 0.5% sodium deoxycholate, 0.1% SDS with protease and phosphatase inhibitors). For western blotting, a rabbit polyclonal phospho-specific antibody against PPARγ Ser273 was produced by AbMax Biotechnology Co., Ltd, China, with a synthetic phosphopeptide previously described^18^. Total tissue lysates were analyzed with anti-PPARγ antibody (Santa cruz).

### Animal experiments

Animal experiments were performed according to procedures approved by the Institutional Animal Use and Care Committee of Xiamen University. 8–10 week-old male mice (*db/*+, *db/db* and KKAy mice from Hua Fukang, Beijing, China) were acclimatized for 7 days under standard conditions before experiments. Mice were fed a high-fat diet (60% kcal fat, D12492, Research Diets Inc, USA) and intraperitoneally (i.p.) injected once daily with vehicle (40% of 2-hydroxypropyl-β-cyclodextrin, HBC) (Sigma, USA), 3 mg/kg of chelerythrine or 3 mg/kg of rosiglitazone for 14 days. Mice were euthanized after 6 h of fasting, and serum samples were collected to measure the metabolic parameters.

### Metabolic parameters analysis

Mice treated with drugs were fasted for 6 h with free access to water. For the glucose tolerance test (GTT), 1 g/kg of glucose was i.p. injected into the mice, and blood glucose was measured with the Accu-Check® Performa (Roche Applied Science, Germany) at 0, 30, 60, 90, and 120 min. For the insulin tolerance test (ITT), 1 units/kg of recombinant human insulin (Novolin 30R, Novo Nordisk, Denmark) was i.p. injected into the mice, and blood glucose was measured at 0, 30, 60, 90, and 120 min after insulin injection. Serum glucose was determined using the Glucose Oxidase Method (APPLYGEN, China), and serum insulin levels were determined by ELISA using an ultra-sensitive mouse insulin kit (Crystal Chem, USA). The cholesterol, Hb1Ac, GSP and ALT levels were assayed using the kits from Nanjing Jiancheng Bioengineering Institute (Nanjing, China).

### Gene expression analysis

Total RNA was isolated from liver and epididymal fat pads using the Tissue RNA kit (Omega Bio-Tek, GA, USA). The RNA was reverse-transcribed using the TAKARA reverse transcription kit. Quantitative PCR reactions were performed with SYBR Green fluorescent dye using a CFXTM96 real-time system (BIO-RAD, USA). Relative mRNA expression was determined by the ΔΔ-Ct method normalized to actin levels. The sequences of the primers are listed in Supplementary Table 2.

## Additional Information

**Accession codes:** The structure of PPARγ complexed with chelerythrine was deposited at the www.pdb.org with PDB ID 4Y29.

**How to cite this article**: Zheng, W. *et al.* Selective targeting of PPARγ by the natural product chelerythrine with a unique binding mode and improved antidiabetic potency. *Sci. Rep.* **5**, 12222; doi: 10.1038/srep12222 (2015).

## Supplementary Material

Supplementary Information
