# Supplementary material for: Selective targeting of PPARγ by the natural product chelerythrine with a unique binding mode and improved antidiabetic potency
Source: Sci Rep. 2015 Jul 17;5:12222. doi: 10.1038/srep12222 (PMC4505335; doi:10.1038/srep12222)
Supplement: Supplementary Information [file srep12222-s1.pdf]

Li et al. Supplementary information
(Supplementary Figure 1, 2, 3, 4 & 5
Supplementary Table 1 & 2)

Selective targeting of PPARγ by the natural product chelerythrine with a unique binding mode and improved antidiabetic potency

Weili Zheng[1], Lin Qiu[1], Rui Wang[1], Xuhui Feng, Yaping Han, Yanlin Zhu, Dezhou Chen, Yijie Liu, Lihua Jin* and Yong Li*

State Key Laboratory of Cellular Stress Biology, Innovation Center for Cell Signaling Network, School of Life Sciences, Xiamen University, Fujian 361005, China

[1]These authors contributed equally to this work

*Address correspondence to Yong Li (yongli@xmu.edu.cn, Tel: 86-592-2181510)

Or Lihua Jin (jinlh@xmu.edu.cn, Tel: 86-592-2181560)

Supplementary Figure 1 Li et al

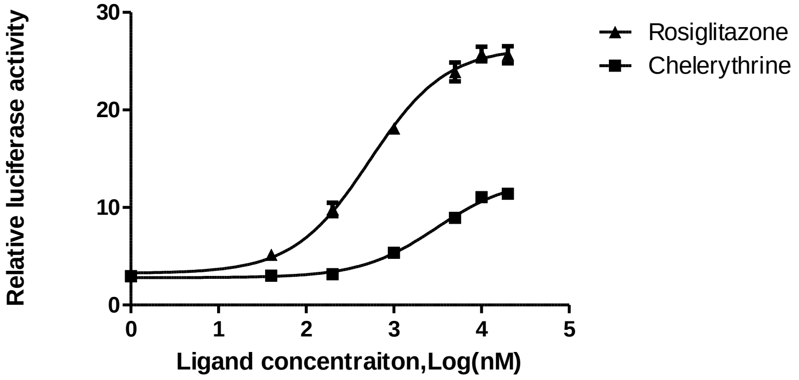

Supplementary Figure 1. Chelerythrine's transcriptional activity on PPARγ.

The gradient concentration of chelerythrine (square symbols) and rosiglitazone (triangle symbols) were used to treat 293T cells for PPARγ-based reporter assay.

Supplementary Figure 2 Li et al

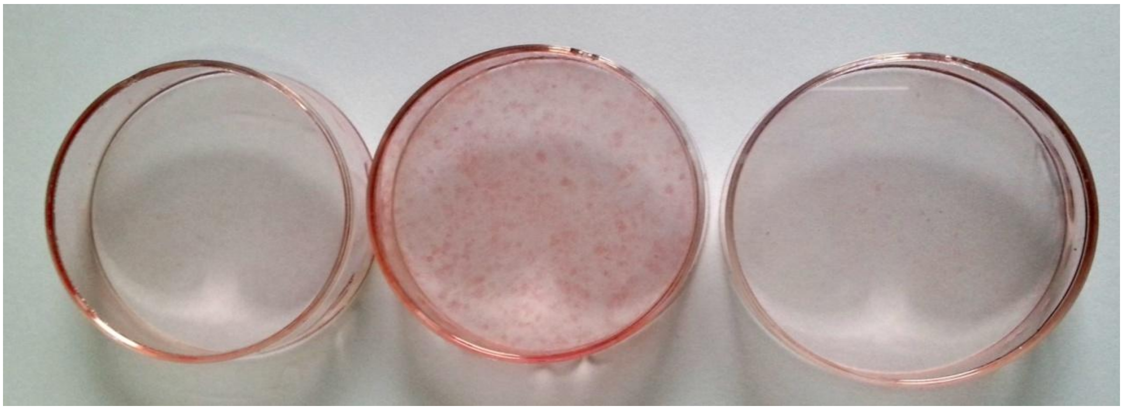

DMSO **Rosiglitazone** **Chelerythrine**

Supplementary Figure 2. Chelerythrine's adipogenesis activity on 3T3-L1 cells.

Oil red O staining of 3T3-L1 cells after treatment for 7 days with DMSO, 10 μmol/l rosiglitazone and 10 μmol/l chelerythrine, respectively. Cells were treated with filtered Oil Red O stock solution [0.5 g of Oil Red O (Sigma, St. Louis,MO) in 100 ml of isopropyl alcohol] for 15 min at room temperature.

Supplementary Figure 3 Li et al

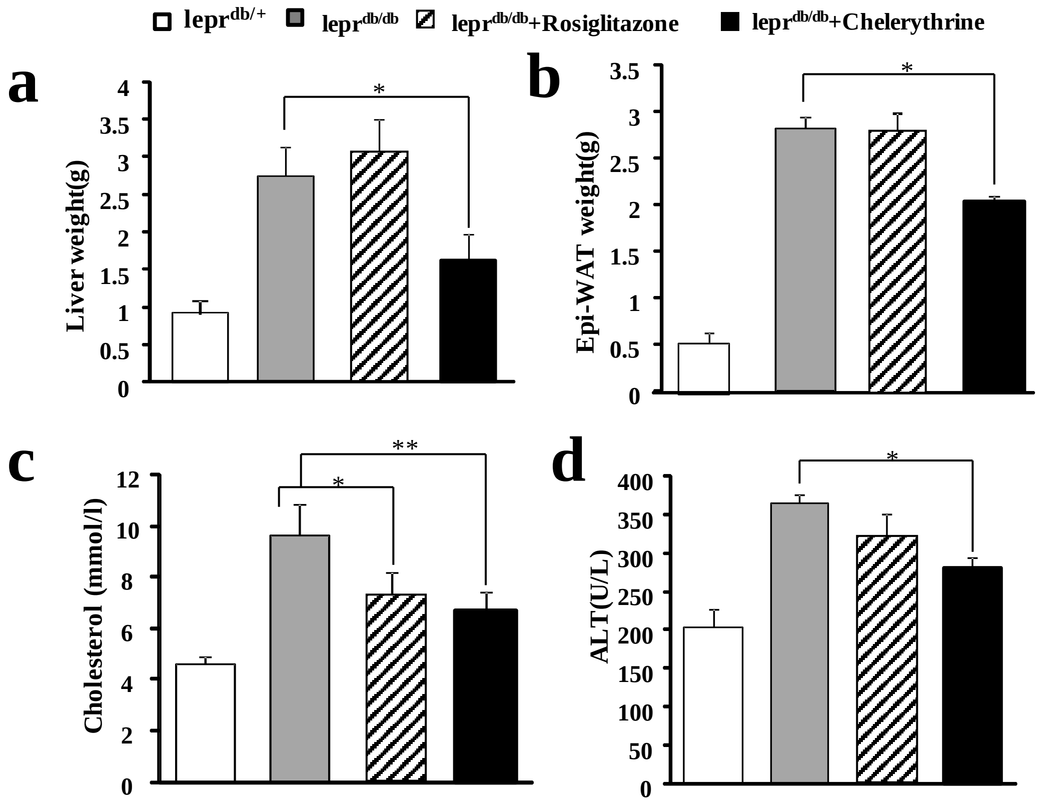

Supplementary Figure 3. The effects of chelerythrine on body weight changes and metabolic parameters.

Liver weight (a), Epi-WAT Weight (b), Cholesterol (c) and ALT (d) in *db/db* mice and their littermate controls (white bars). *db/db* mice were i.p. injected with vehicle (HBC, Gray bars), 3 mg/kg rosiglitazone (Rosi, stripped bars) and 3 mg/kg Chelerythrine (CHE, black bars) for 14 days. Error bars represent s.e.m.; $^{*}p<0.05$, $^{**}p<0.01$ compared to vehicle treated *db/db* mice .

Supplementary Figure 4 Li et al

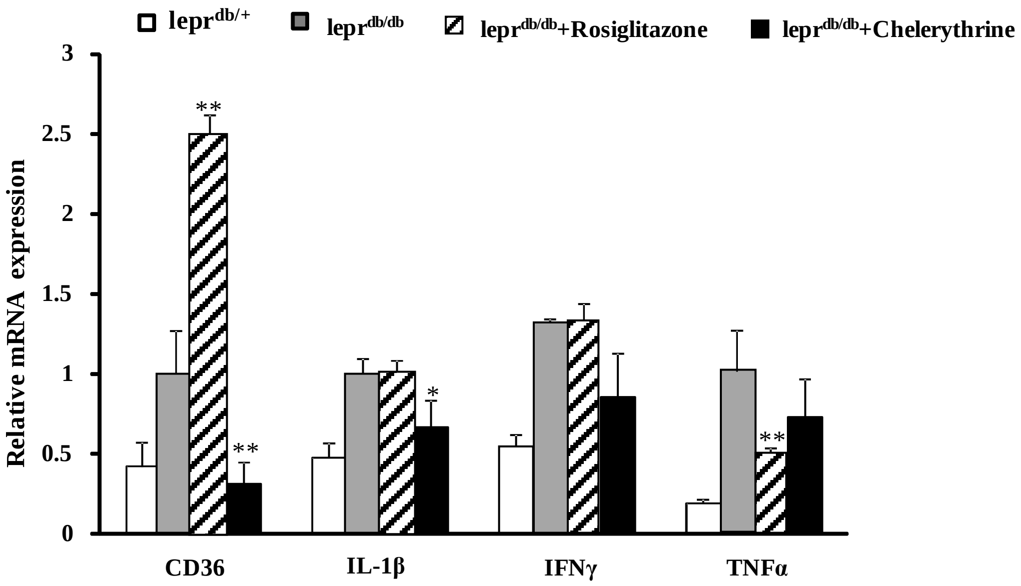

Supplementary Figure 4. Cheletythrine improved the inflammation genes in epi-WAT.

The mRNA levels of genes associated with inflammtion process were analyzed in epididymal fat pads of *db/db* diabetic mice and their littermate controls (white bars). *db/db* mice were i.p. injected with vehicle (HBC, Gray bars), 3 mg/kg rosiglitazone (Rosi, stripped bars) and 3 mg/kg Chelerythrine (CHE, black bars) for 14 days. Error bars represent s.e.m.; $^{*}p<0.05$, $^{**}p<0.01$ compared to vehicle treated *db/db* mice .

Supplementary Figure 5 Li et al

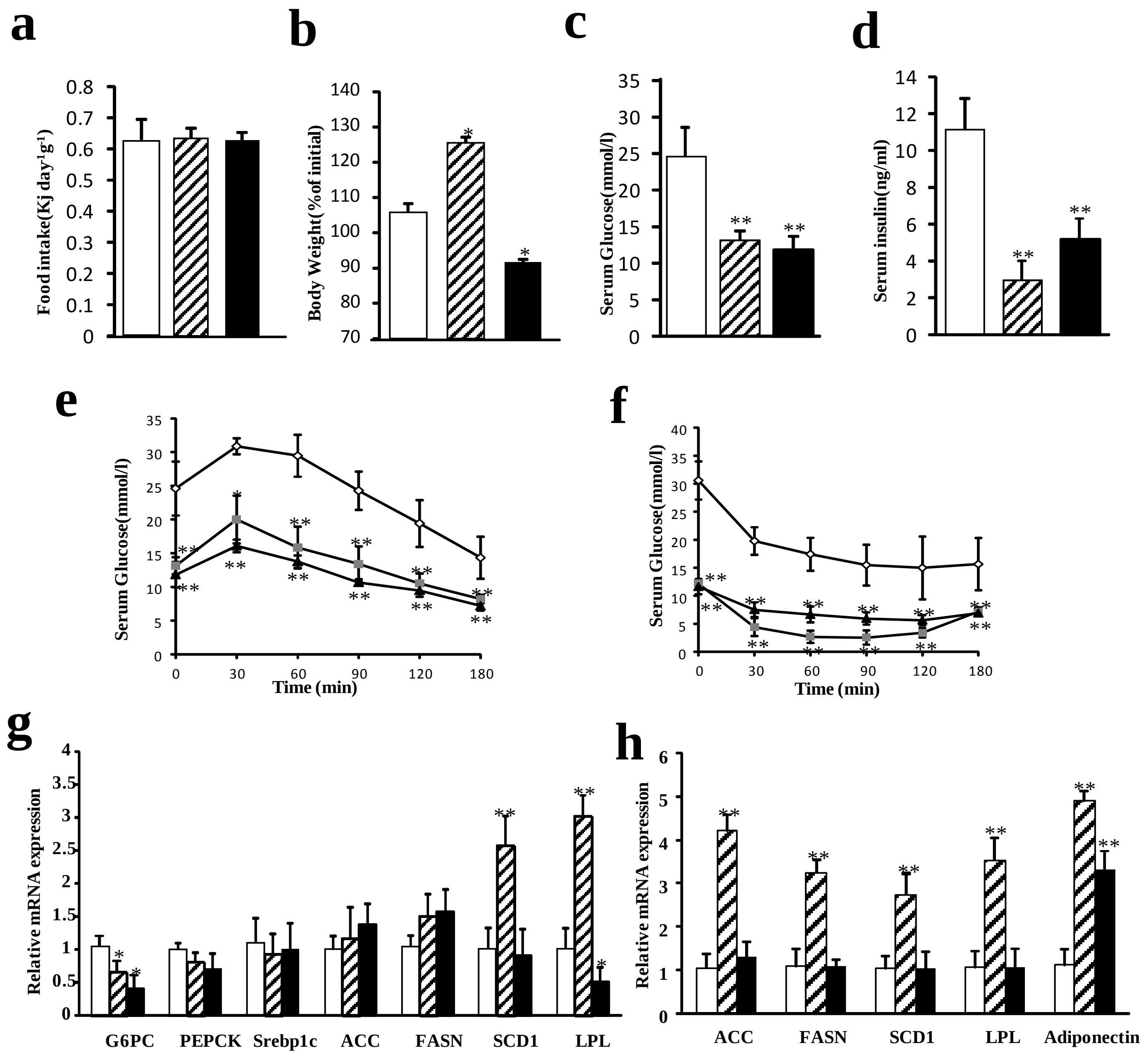

Supplementary Figure 5. Chelerythrine improved glucose tolerance and insulin sensitivity with reduced adipogenesis activity in KKAy mice. The food intake (a), body weight (b), serum glucose (c) and insulin (d) levels were measured after KKAy mice were i.p. injected with vehicle (HBC, white bars or diamond symbols), 3 mg/kg rosiglitazone (Rosi, stripped bars or square symbols) or 3 mg/kg chelerythrine (CHE, black bars or triangle symbols) for 14 days. Insulin (1 unit/kg) and glucose (1 g/kg), respectively, were administered by i.p. injected in 6-h-fasted KKAy mice for the GTT (e) and ITT (f). (g-h) The mRNA of genes involved in adipogenesis and glucose homeostasis in liver (g) and epididymal fat pads (h). Error bars represent s.e.m.; *p<0.05, **p<0.01 compared to vehicle treated KKAy mice.

Supplementary Table 1

Data collection and refinement statistics

	PPARγ/Chelerythrine
**Data collection**	
Space group	P1211
Cell dimensions	
a, b, c (Å)	43.62, 54.51, 66.40
α, β, γ (°)	90.00, 107.07, 90.00
Resolution (Å)	50-1.98(2.01-1.98)*
Rsym	0.068(0.356)
I / σ	24.8(4.0)
Completeness (%)	99.9(100)
Redundancy	5.3(5.2)
**Refinement**	
Resolution (Å)	41.36-1.97
No. reflections	30654
R_{work} / R_{free}	16.2/20.1
No. atoms	
Protein	2144
Ligand/ion	26
Water	207
B-factors	
Protein	28.824
Ligand/ion	63.085
Water	32.968
R.m.s. deviations	
Bond lengths (Å)	0.0196
Bond angles (°)	2.1209

*Values in parentheses are for highest-resolution shell.

Supplementary Table 2

The sequences of primers used in quantitative PCR.

Genes	Primer sequences
Srebp1c	F: GGTCAAAACCAGCCTCCCA
	R: CAGTCCCCGTCCACAAAGA
Acc	F: CCCAGCAGAATAAAGCTACTTTGG
	R: TCCTTTTGTGCAACTAGGAACGT
Fasn	F: TGCTCCCAGCTGCAGGC
	R: GCCCGGTAGCTCTGGGTGTA
SCD1	F: ACCTGCCTCTTCGGGATTTT
	R: GTCGGCGTGTGTTTCTGAGA
G6PC	F: ATGGAGGAAGGAATGAACA
	R: TGGGAAAGAGGACATAGAA
Adiponectin	F: GAATCATTATGACGGCAGCAC
	R: CCAGATGGAGGAGCACAGAG
Adipsin	F: CATGCTCGGCCCTACATGG
	R: CACAGAGTCGTCATCCGTCAC
LPL	F: TGAAAGTGGGTTTTCCTGAGTAT
	R: GGTTAGCCACCGTTTAATATTTG
IFNγ	F: GCTTTGCAGCTCTTCCTCAT
	R: TTTGCCAGTTCCTCCAGATA
IL-1β	F: AGAGCATCCAGCTTCAAATC
	R: GCTTCTCCACAGCCACAAT
TNFα	F: TCTCATTCCTGCTTGTGGC
	R: CACTTGGTGGTTTGCTACG
CD36	F: CCTCTTCCCGTCAGACTTGTG
	R: TCGGAGGTGTTGGCTAGTATTT
